# Comparative Hepatotoxicity of Aflatoxin B1 among Workers Exposed to Different Organic Dust with Emphasis on Polymorphism Role of Glutathione S-Transferase Gene

**DOI:** 10.3889/oamjms.2016.051

**Published:** 2016-04-20

**Authors:** Amal Saad-Hussein, Eman M. Shahy, Weam Shaheen, Mona M. Taha, Heba Mahdy-Abdallah, Khadiga S. Ibrahim, Salwa F. Hafez, Nevein N. Fadl, Karima A. El-Shamy

**Affiliations:** 1 *Department of Environmental & Occupational Medicine, National Research Centre, Cairo, Egypt*; 2 *Department of Medical Physiology, National Research Centre, Cairo, Egypt*

**Keywords:** Organic dust, Aflatoxin B1, Liver enzymes, GST polymorphism

## Abstract

**AIM:**

The study aimed to investigate effects of organic dust exposure from different sources on aflatoxin B1-albumin adducts (AFB1/Alb), and role of glutathione S-transferase (GST) gene polymorphism in hepatotoxicity of (AFB1) among exposed workers.

**MATERIAL AND METHODS:**

Liver enzymes, AFB1/Alb, and GST polymorphism were estimated in 132 wheat flour dust and 87 woods sawmill workers, and 156 controls.

**RESULTS:**

Results revealed that AFB1/Alb and liver enzymes were significantly elevated in exposed workers compared to controls, and were significantly higher in sawmill workers compared to flour workers. AFB1/Alb in flour and sawmill workers with GSTT1 and GSTM1&GSTT1 null genotypes were significantly higher than controls, and in sawmill workers with GSTM1&GSTT1 null than flour workers. Liver enzymes (ALT and AST) in sawmill workers were significantly higher than flour workers and controls in all GST polymorphism; except in GSTT1 polymorphism, where these enzymes were significantly higher in the two exposed groups than controls.

**CONCLUSIONS:**

In conclusion, organic dust exposure may cause elevation in AFB1/Alb and liver enzymes of exposed workers, and GST gene polymorphism plays an important role in susceptibility to hepatic parenchymal cell injury; except in workers with GSTT1&GSTM1 null genotype, gene susceptibility seemed to have little role and the main role was for environmental exposures.

## Introduction

Organic dust may contain bacteria and its` endotoxins, fungi and its` mycotoxins, viruses, high molecular weight allergens, plant fibres, pollen, peptidoglycans, β (1→3)-glucans, etc. Therefore, exposures to organic dust contaminated with biological agents in occupational indoor environment are associated with a wide range of adverse health effects, including infectious diseases, allergies, toxic effects and cancer [1]. Liebers *et al* [2] reported that endotoxins are the main constituents of organic dust.

Mycotoxins of *Aspergillus, Penicillium* and *Fusarium* genera are known to be present in the inhalable fraction of airborne corn dust [3], cotton dust [4-5], grain dust [6-7] and wood dust[8]. Genetic predisposition of the mould to produce mycotoxins, substrate, humidity and the presence of fungicides or other competitive microbial species are factors which cause mycotoxin production in vitro [9].

Many studies have demonstrated the association between the ingestion of aflatoxin-contaminated foods and its hepatotoxic effects [10-11]. Yet few studies have measured the toxicity among people occupationally exposed to aflatoxin. In a registry based analysis of occupational risks for primary liver cancer in Sweden, significant excesses were observed in both male and female workers in grain mills. This finding was associated with potential exposures to the hepatotoxins, aflatoxins, parasites, pesticides, and fumigants [12].

Workers exposed to organic dust in closed areas are often subjected to very high levels of microorganisms and its’ toxins. High airborne counts of *Penicillium* and *Aspergillus* (mainly *Aspergillus niger* and *Aspergillus flavus*) were detected in the different departments in textile industry and were accompanied with elevation in the aflatoxin M1 (AFM1) the metabolite of aflatoxin B1 (AFB1) in the textile workers compared to their controls [5]. There was also increased risk of elevation in serum AFB1 in workers exposed to *Aspergillus* in the working environment; such as in bakers and wheat flour mill workers [7]. Such elevation in the aflatoxins has potent hepatotoxic and hepatocarcinogenic effects [13, 14].

Aflatoxin B1-albumin adduct (AFB1/Alb) is the major protein adduct found in peripheral blood due to exposure to aflatoxin B1. The use of AFB1/Alb; as a biomarker for aflatoxin exposure, has several advantages: (1) AFB1/Alb reflect DNA damage in hepatocytes, as does aflatoxin-N7-guanine in urine; (2) AFB1/Alb, at least in experimental animals, are as long-lived as albumin, which has a half-life of twenty-one days in humans, and thus provide a measure of exposure over a period of two -three months; and (3) multiple measurements of urinary aflatoxin are required to reflect average exposure, but only a single measurement of AFB1/Alb is needed to provide a representative average exposure.

In other words, serum level of AFB1/Alb is a better estimation of long-term biologically effective dose of aflatoxin exposure than in urinary level of aflatoxin- N7-guanine [15].

The increasing focus on mycotoxins, particularly in the grain production industry, along with unavoidable dust exposure during crop handling, have led to a growing concern about the inhalable contribution of mycotoxin exposure in occupational settings [7, 16]. Cell susceptibility to mycotoxins mainly depends on mycotoxin concentrations, duration of exposure, and intracellular molecular and genetic context [17].

Glutathione S-transferase genes (GST) constitute an enzymatic super family of phase II iso-enzymes that protect against endogenous oxidative stress, as well as exogenous potential toxins. “GSTs” detoxify a variety of electrophilic compounds, including oxidized lipid and DNA products generated by reactive oxygen species damage to intracellular molecules. The two isoforms that are most extensively studied are GST Mu1 (*GSTM1*) gene and GST Theta1 (*GSTT1*) gene since their activity is modulated by genetic polymorphisms, they may regulate an individual’s ability to metabolize the ultimate carcinogen of aflatoxins, the epoxide [18].

The *GSTM1* and *GSTT1* genes frequently have a partial deletion that causes absence of enzymatic function. Both polymorphic traits are inherited independently. Homozygotes for the mutated inactive alleles of each gene are devoid of any specific enzymatic activity in all tissues (null genotypes). Available evidence indicates that GST isozymes, including the *M1* and *T1* isoforms, are involved in the metabolism of a wide range of carcinogens. This provides a biological basis for a putative role of GST polymorphisms in the risk of developing cancers related to these substances. Indeed, *GSTM1* and *GSTT1* polymorphisms have been considered as risk factors for developing tumors such as lung cancer and hepatocellular carcinoma [19].

The present work aimed at comparing AFB1/Alb adduct in workers exposed to organic dust from different sources (wheat and wood dust), and the role of GST gene polymorphism in development of the hepatotoxic effects of AFB1.

## Methodology

### 

#### Subjects

Cross-sectional comparative study was conducted. The study was performed among 132 workers exposed to wheat flour dust (flour workers); 81 wheat mill and 51 bakery workers, 87 workers exposed to wood dust (sawmill workers), and 156 control workers not occupationally exposed to organic dust (controls). The controls were comparable with the industrial workers for the confounding factors; age and smoking habits. All the included workers were exposed for more than 5 years. Ethical approval was taken from the Ethical Committee of National Research Centre, and a written consent was obtained from all the included subjects.

The flour workers are exposed to wheat flour dust during the milling processes; by which wheat is ground into flour. The included workers were garbling workers (43 workers), grinding workers (15 workers) and packaging workers (23 workers). Garbling workers are exposed to wheat dust while carrying and evacuating the wheat bales.

Grinding workers are exposed to wheat dust during sequencing, breaking, grinding and separating the bran and germ from the endosperm. The last step is packing of the different milled products to be ready for marketing (Packaging workers). Bakers (51 workers) are exposed to wheat flour dust during the process of baking while mixing of the flour, water, salt and yeast to prepare for the dough fermentation step.

The sawmill workers are exposed to wood dust during selecting the timber bundle and during cutting of each timber into the required lengths, as well as during bundling of the cut timbers and processing the shapes and sizes required.

#### Questionnaire

After obtaining the managerial approvals in each factory, and the written consents from the included workers and the control subjects, a questionnaire was completed through personal interview conducted by the medical staff of the project. The questionnaire included detailed personal, medical, occupational and environmental histories.

#### Blood sampling

About 5 ml venous blood sample was collected from each participant during their working shifts and divided in 2 tubes, 2 ml blood on EDTA tube which was kept frozen at -20°C for screening of “GST” polymorphisms. And 3 ml in another dry sterile tubes, left to clot for 30 minute at 37°C and then centrifuged at 3,000 rpm for 10 minutes, and the sera were kept at -20°C.

#### Determination of Aflatoxin B1 “AFB1” level in serum

Aflatoxin B1 was firstly extracted from the serum using EASI-EXTRACT®Aflatoxin-immunoaffinity column. Easi-Extract® aflatoxin immunoaffinity columns (IACs) (cat. # RP70N) were purchased from R-Biopharm (Glasgow, Scotland). The extracted sample was applied to the ELISA according to RIDASCREEN®Aflatoxin B1 ELISA kit.

#### Determination of Serum Albumin (Alb)

Serum albumin was determined by colorimetric method according to Doumas *et al*. [20].

#### Determination of liver enzymes

Serum aspartate aminotransferase “AST” and alanine aminotransferase “ALT” were determined according to the colorimetric method described by Reitman & Frankel [21] using bio-diagnostic kits (www.bio-diagnostic.com).

Serum Alkaline phosphatase “ALP” was determined according to the colorimetric method based on that described by Belfield & Goldberg [22] using bio-diagnostic kits (www.bio-diagnostic.com).

Screening of glutathione-S-transferase “GST” polymorphisms (GSTM1, GSTT1 genotypes) using polymerase chain reaction PCR.

#### DNA extractions

DNA was extracted from whole blood sample using Genomic DNA Purification kit (Gene JET™/Fermentas).

#### PCR amplification

The “GSTM1” and “GSTT1” genotypes were determined by co-amplification of both genes with PCR. Briefly [23], PCR was performed in a 25-ml mixture containing the buffer supplied by Promega (Madison, WI, USA), 250 ng genomic DNA, Taq DNA polymerase (1 U), four bases (dNTP) and 200 μg of each primer. The primers used for the GSTM1 gene were 5’-CTGCCCTACTTGATTGATGGG-3’ and 5’-CTGGATTGTAGCAGATCATGC-3’. The primers used for the GSTT1 gene were 5’ -TTCCTTACTGGTCCTCACATCT C-3’ and 5’-TCACC-GGATCATGGCCA GCA-3’. The human B-globin gene (110 bp) was also amplified in each reaction as a positive control to confirm the presence of amplifiable DNA in the samples.

The primers used for B-globin were 5’-ACACAACTGTGTTCACTAG-C-3’ and 5’-CAACTCATCCACGTTCACC-3’.

The amplification was carried out in 35 cycles with denaturation at 94°C for 1 min 30 s, annealing at 52°C for 1 min, and extension at 65°C for 1 min. The PCR products were then resolved by electrophoresis in 2% agarose gels, stained with ethidium bromide and photographed under ultraviolet light. Individuals with one or more GSTM1 alleles had a 273-bp fragment, and individuals with one or more GSTT1 alleles had a 480-bp fragment.

#### Statistical analysis

The collected data were statistically analyzed using SPSS package version 18. Quantitative data were represented as mean ± standard deviation (SD). Quantitative comparisons with normal distribution were done through Analysis of Variance (ANOVA) and Least Significant difference (LSD) for more than two comparable groups. Chi-square was used to compare qualitative results. The relationships between the different variable were studied through correlation coefficient. The difference was considered significant at P-value ≤ 0.05 levels.

## Results

All the included subjects in this study were males. There was no significant difference in the age of flour workers (45 ± 8.9 years), sawmill workers (48 ± 7.8 years), and controls (44 ± 9.2 years) (F-ratio = 1.45, P > 0.05). Smoking habits showed no significant difference between the three studied groups (Chi-square = 0.33, P > 0.05). The workers were employed for more than 5 years (flour workers was 15 ± 5.2 years, and sawmill workers was 17 ± 5.3 years), with no significant difference in the duration of exposure between the different occupationally exposed groups (t = 0.44, P > 0.05).

Table 1 showed that AFB1/Alb was significantly elevated in the flour and sawmill workers compared to the control workers, and was significantly higher in the sawmill workers compared to the flour workers. The liver enzymes AST, ALT and ALP of the workers in the two studied industries were significantly higher compared to their controls. AST and ALT were significantly elevated in the sawmill workers compared to the flour workers.

**Table 1:** Comparison of AFB1/Alb and liver enzymes of the workers with different exposures and their controls

		N	Mean	SD	ANOVA	

					P-value	LSD
AFB1/Alb (ng/g)	Controls	78	0.04	0.002	< 0.0001	(f,s)
	
	Flour workers	132	0.07	0.004		(c,s)
	
	sawmill workers	87	0.10	0.005		(c,f)

AST (U/L)	Controls	78	16.71	1.931	< 0.0001	(f,s)
	
	Flour workers	132	25.00	0.812		(c,s)
	
	sawmill workers	87	30.99	0.640		(c,f)

ALT (U/L)	Controls	78	22.54	3.158	< 0.0001	(f,s)
	
	Flour workers	132	30.76	1.118		(c,s)
	
	sawmill workers	87	41.54	0.804		(c,f)

ALP (IU/L)	Controls	78	65.34	4.089	< 0.005	(f,s)
	
	Flour workers	132	80.44	2.597		(c)
	
	sawmill workers	87	80.23	4.329		(c)

N.B. c = controls, f = workers exposed to flour dust, and s = sawmill workers

However, AFB1/Alb and the liver enzymes were not significantly associated with the duration of exposures in the workers from the two industrial groups. Table 2 showed that the liver enzymes AST and ALT were significantly correlated with the AFB1/Alb levels in the examined three groups, while, ALP was not associated with AFB1/Alb in the three groups.

**Table 2:** Relationships between AFB1/Alb and the liver enzymes in the examined workers

	AFB1/Alb (ng/g)			

	Flour workers (136)		Sawmill workers (156)	

	r =	P-value	r =	P-value
AST (U/L)	0.3	< 0.03	0.3	< 0.05

ALT (U/L)	0.4	0.001	0.5	< 0.005

ALP (U/L)	0.1	NS	0.04	NS

In the different GST polymorphisms, AFB1/Alb in the sawmill workers was higher than the flour workers and the controls. In *GSTM1 & GSTT1* null genotype, AFB1/Alb in sawmill and flour workers were significantly higher compared to the controls, and in the sawmill workers than in the flour workers. While, AFB1/Alb in flour and sawmill workers with *GSTT1* genotype were significantly higher than in the controls. There was no significant difference detected in the AFB1/Alb between the three examined groups in both those with *GSTM1* and those with *GSTM1&GSTT1* genotype (Table 3). Moreover, there was no significant difference in AFB1/Alb between the different GST genotypes in each of the three examined groups. But, AFB1/Alb adduct seemed to be the highest in the subjects with *GSTT1* in the three examined groups.

**Table 3:** Comparison of AFB1/Alb and liver enzymes of the workers with different exposures compared to the controls according to GST gene polymorphism

		Null		GSTM1		GSTT1		GSTT1&GSTM1	

		Mean	SD	Mean	SD	Mean	SD	Mean	SD
AFB1/Alb (ng/g)	Controls	0.04 (f,s)	.003	0.04	0.01	0.05 (f,s)	0.001	0.05	0.01

	Flour workers	0.07 (c,s)	.004	0.07	0.01	0.08 (c)	0.002	0.05	0.01

	sawmill workers	0.10 (c,f)	.005	0.09	0.01	0.13 (c)	0.01	0.09	0.02

	P-value	< 0.0001		> 0.05		< 0.05		> 0.05	

AST (U/L)	Controls	20.8 (s)	2.1	14.0 (s)	0.4	16.5 (f,s)	1.5	16.0 (s)	2.0

	Flour workers	25.7 (s)	1.0	20.8 (s)	1.7	27.7 (c)	2.6	18.3 (s)	3.8

	sawmill workers	32.4 (c,f)	1.0	30.4 (c,f)	1.1	29.3 (c)	2.6	27.7 (c,f)	1.4

	P-value	< 0.0001		< 0.0001		< 0.005		< 0.0001	

ALT (U/L)	Controls	29.3 (s)	3.3	21.6 (s)	0.2	31.0 (f,s)	0.01	14.5 (s)	0.5

	Flour workers	34.0 (s)	1.4	26.3 (s)	1.9	35.5 (c)	4.6	19.2 (s)	6.1

	sawmill workers	43.6 (c,f)	1.2	39.3 (c,f)	1.8	39.3 (c)	2.5	40.7 (c)	2.0

	P-value	< 0.0001		< 0.0001		0.001		< 0.0001	

ALP (IU/L)	Controls	82.3	5.1	78.0	13.6	69.0	19.0	69.5	7.5

	Flour workers	78.1	3.3	78.4	3.6	80.5	10.1	62.5	9.2

	sawmill workers	68.1	6.1	67.9	9.0	63.0	11.0	65.8	15.9

	P-value	> 0.05		> 0.05		> 0.05		> 0.05	

N.B. c = controls, f = workers exposed to flour dust, and s = sawmill workers.

Table 3 also revealed that the liver enzymes AST and ALT in the sawmill workers were significantly higher than in the flour workers and the controls in all the GST polymorphism; except in the workers with *GSTT1*. Among workers with *GSTT1*, there was no significant difference between sawmill and flour dust workers, and the two workers groups were significantly higher compared with their controls. There was no significant difference in the ALP between the three groups in the four GST polymorphism.

## Discussion

Aflatoxins are metabolites of *Aspergillus* genera which are widespread in the natural environment [24]. They are the most dangerous metabolites with high risk to cause human hepatocellular carcinoma (HCC) [25-27]. Although there are many confounding factors that can influence liver functions, aflatoxin is one of the most causative pollutant that affect liver functions [28].

The level of AFB1/Alb in peripheral blood is a reliable indicator of long-term exposure to aflatoxin [29]. The present study found that AFB1/Alb in the workers exposed to organic dust (flour and sawmill workers) was significantly higher compared to their controls. Moreover, AFB1/Alb was also significantly higher in sawmill workers than flour workers.

Previous study revealed that the elevation in AFB1/Alb serum levels were compatible with the elevation of *Aspergillus* *spp*. (fungi producing AFB1) in the working environment of bakeries and wheat mill (487.2 CFU/m^3^ and 198.9 CFU/m^3^ respectively) compared to the environment of the controls (12.8 CFU/m^3^) [7]. In sawmill factories, Klarić *et al* [30] detected that *Penicillium spp*. was the dominant fungal genera which occurred in 50-100% of the environmental samples.

While, there was seasonal variations in the occurrence of *Aspergillus spp.;* *Aspergillus niger* peaked in April-May (71% and 65%), while *Aspergillus flavus* was detected with a relatively low frequency (2%-15%) over the whole period of the year, and *Aspergillus fumigatus* was recovered with the highest frequency in November-December (17% and 30%). In Egypt, fungi levels were recorded in wood working shops at mean values of 10^3^ and 10^2^ CFU/m^3^, *Penicillium*, *Aspergillus*, and *Cladosporium* were the predominant fungi all over the year [31]. That could explain the significant elevation in the AFB1/Alb adducts detected in the serum of the flour and sawmill workers in the present study.

In a previous study, significant correlation between AFB1/Alb and the duration of exposure in bakers was detected but not in the wheat mill workers [7]. The environmental concentrations of *Aspergillus flavus* were compatible with those results. The non-significant association of AFB1/Alb and the liver enzymes with the duration of exposure in the present study could be attributed to inter-individual variation in the activity of liver enzymes responsible for AFB1 metabolism, that predispose to individual response to AFB1 toxicity. Genetic susceptibility could have an important role in the response to environmental toxins. Therefore, GST polymorphism (the gene responsible for detoxification of AFB1) was studied in the present study.

The polymorphism of GST revealed that there was no significant effect on the levels of AFB1/Alb adducts between the workers with the different GST genotypes in each of the three examined groups. But, AFB1/Alb adduct seemed to be the highest in the subjects with *GSTT1* in the three examined groups. Chen *et al* [32] stated that subjects with *GSTT1* genotype may be genetically predisposed for increased cancer risk due to aflatoxins, and Saad-Hussein *et al* [33] found that serum AFB1/Alb levels were significantly higher in flour mill workers with *GSTT1* compared to the workers with the other GST genotypes. They concluded that wheat flour handlers with *GSTT1* genotype have less ability to detoxify AFB1 that leads to elevation in their serum AB1/Alb adduct.

In the present study, however each of the flour and sawmill workers were exposed to the same workplace environmental conditions, workers with *GSTT1* genotype could not detoxify AFB1 in the same rate of the detoxification of AFB1 in workers with *GSTM1*; whether homozygous genotype or heterozygous genotype (*GSTM1&GSTT1*). This could be proved through the loss of significant difference between the two groups of workers exposed to organic dust with both *GSTM1* and *GSTM1&GSTT1* genotypes and their controls. While among the workers with *GSTT1*, there was significant elevation of AFB1/Alb adduct in the flour and sawmill workers with *GSTT1* compared to their controls, but, there was no significant difference in AFB1/Alb adduct levels between the flour and sawmill workers.

In population with *GSTT1&GSTM1* null genotype, gene susceptibility seemed to have little role and the main role was for environmental exposures, as there was significantly elevation in AFB1/Alb adduct in sawmill and flour workers with *GSTT1&GSTM1* null genotype compared to their controls, and in the sawmill workers compared to flour workers.

Aflatoxin B1 is a cytotoxic and carcinogenic mycotoxin, and the liver is considered the primary target of AFB1 toxicity [34]. Serum liver enzyme activities are valuable tools for detecting liver injury. Elevation in liver enzymes suggests either hepatic parenchymal cell injury (ALT and AST) or biliary tract alterations (ALP) [35]. In the current study, the liver enzymes AST, ALT and ALP of the workers in the two studied industries were significantly higher compared to their controls. This could be attributed to the elevation of their AFB1/Alb compared to the controls; as AST and ALT were significantly correlated with the AFB1/Alb levels. Several studies were agreed with the present results, which detected an increase in AFB1/Alb levels and liver enzymes among Chinese population [36], Ghanaians population [37], and Egyptian wheat flour handlers [7, 14].

However ALP was significantly increased among the flour and sawmill workers in the current study compared to their controls, it was not significantly correlated with their AFB1/Alb. This means, that workplace environmental pollution causes significant elevation in the AFB1/Alb adducts that could have significant effect on the liver parenchyma of the exposed workers rather than the effects on their biliary tracts. This significant effect was not related to their duration of exposures; as the liver enzymes and AFB1/Alb were not significantly associated with the duration of exposure. Similarly, Higashi *et al* [38] revealed that both ALT and AST were significantly correlated with aflatoxin-albumin. Also, Peng *et al* [39] found that ALT and AST were significantly correlated with AFB1/Alb in Chinese population, while, Wild *et al* [40] found that serum ALT levels were significantly correlated with aflatoxin-albumin levels.

The liver enzymes ALT and AST in the current study were significantly elevated in sawmill and flour workers with *GSTT1* genotype compared to their controls. These elevations could be attributed to the suspected decrease in the activities of the detoxification enzymes in those exposed groups. While, ALT and AST in the sawmill workers with *GSTM1&GSTT1* null genotype, with *GSTM1* genotype, and *GSTM1&GSTT1* were significantly elevated compared to the controls and the flour workers. This could be attributed to the high occupational exposure to wood dust that may contain high concentrations of *Aspergillus* as previously proved [30, 31], and not to gene susceptibility.

In conclusion, organic dust exposure in the workplace may cause elevation in AFB1/Alb and liver enzymes in the exposed workers, and this elevation depends on the type of dust. Workers with *GSTT1* genotype were found to have susceptible risk of hepatic parenchymal cell injury due to exposure to aflatoxins. Hence, GST gene polymorphism plays an important role in prediction of susceptibility to the hazardous effects of environmental exposure AFB1, except among workers with *GSTT1&GSTM1* null genotype, gene susceptibility seemed to have little role and the main role was for environmental exposures. Further studies are recommended.
